# Metabolites profiling reveals the dynamic changes of non-volatiles in Pu-erh during Ganpu tea processing

**DOI:** 10.1016/j.fochx.2023.100774

**Published:** 2023-06-26

**Authors:** Xinyi Deng, Shiqiang He, Yuxin Han, Yingjuan Chen

**Affiliations:** Department of Tea Science, College of Food Science, Southwest University, Chongqing 400715, China

**Keywords:** Pu-erh tea, Citrus peel, Non-volatiles changes, Fixation process

## Abstract

Ganpu is an unique tea product made by Pu-erh tea and citrus peel. In this study, the non-volatiles changes of Pu-erh during Ganpu tea processing were fully analyzed by UPLC–ESI-MS/MS. Total 276 significantly differential metabolites in Pu-erh during Ganpu processing were detected (P < 0.05, VIP > 1), and their change trend were clustered into 8 subclasses by K-means analysis. Metabolites of Pu-erh present at various processes were revealed. 72 differential metabolites (P < 0.05, VIP > 1 and fold change ≥2 or ≤0.5) between any two stages were identified and fixation was the key step with 61 differential metabolites. 39 flavonoids and 2 lignans and coumarins were significantly decreased after fixation, while 5 terpenoids, 3 amino acids, 1 organic acids, 2 nucleotides and derivatives and newly detected jasminoside A (Log_2_FC = 9.90), picrocrocin (Log_2_FC = 9.90) and nomilinic acid (Log_2_FC = 7.56) were significantly increased. The results provided valuable information about the effect of Ganpu processing on dynamic changes of non-volatiles in Pu-erh.

## Highlights

• Ganpu tea made by *Citrus reticulate* Chachi peel and Pu-erh ripen tea were studied.
• Dynamic changes of non-volatiles in Pu-erh during processing were fully revealed.
• Fixation was the key step with 61 differential metabolites, and most were flavonoids.
• Three newly detected metabolites were most significantly increased after fixation.
• Amino acids and soluble sugars content were similar, but compositions were different.

## Introduction

Pu-erh tea, made by tea leaves of a large leaf tea species (*Camellia sinensis* var. *assamica*) in Yunnan province, China, is consumed all over the world and popular for its various health-promoting effects such as anti-inflammatory, antioxidation, anti-aging and hypolipidemic efficacies, and weight-loss activities (Kuo et al., 2005). Pu-erh tea is classified as Pu-erh ripen tea and Pu-erh raw tea based on the processing techniques and unique flavor characteristics. Pu-erh ripen tea is a kind of fermented tea, which is made from sun dried green tea by microbial fermentation (Kuo et al., 2005, Lv et al., 2013). Ganpu tea, originated from Tang dynasty, is an unique tea product made by Pu-erh ripen tea and citrus peel. Ganpu possessing the characteristics of both citrus pericarp and Pu-erh tea is increasingly favored by consumers in the Chinese tea market due to the potential health benefits and flavor characteristics (Lv et al., 2013, Yu et al., 2018). The flavor of Ganpu tea is mainly dependent on several factors, such as citrus cultivars, Pu-erh tea resources, and processing technologies. Currently, in the tea market, there are different kinds of Ganpu tea which are made by different citrus cultivars with different maturity (mature, near mature and immature) and various kinds of tea materials (dark tea “Tianjian”, Pu-erh raw tea and ripened Pu-erh tea) (Qi et al., 2020, Xu et al., 2021, Xiao et al., 2021). In terms of raw materials, Pu-erh tea and *Citrus reticulate* Chachi pericarp are commonly used to produce Ganpu tea. At present, Pu-erh ripen tea mainly made in Yunnan province of China and *C. reticulate* Chachi widely planted in Xinhui county, Guangdong province of China (Zheng et al., 2018) are the raw materials for Ganpu tea production, which not only contains both characteristics but also mixes the citrus flavor with the taste of Pu-erh ripen tea. The pericarp of citrus are rich in many kinds of enzymes and functional components, which can catalyze the transformation of major compounds during the process of Ganpu tea, providing Ganpu tea with fruity flavor and citrus aroma (Jeong et al., 2022). Furthermore, the pericarp of citrus has rich phenolic acids and flavonoids which has considerably higher antioxidant activity than the fruit of citrus. Dried citrus peel (Chen-pi) is well known for its health benefits, which has been used as traditional medicine in China for relieving cough and reducing sputum (Jeong et al., 2022).

In recent years, metabolomic methods have been widely used to comprehensively identify the non-volatiles and volatiles present in tea. The volatile organic compounds of Ganpu tea mainly include olefins, esters and alcohols (Jeong et al., 2022, Qi et al., 2020). Qi et al. (2020) analyzed the aromatic substances of Ganpu tea (produced from citrus peel and dark tea “Tianjian” from Anhua county, Hunan province, China) by headspace solid-phase microextraction-gas chromatography-mass spectrometry (HS-SPME-GC–MS) and a number of volatile compounds were identified. Sun drying is an usually applied procedure in the processing of some Ganpu tea. The transformations of chemical metabolites of Ganpu tea during the sun drying processing were studied by Zheng et al. (2020), and total 92 metabolites were identified in the hot-water extracts of Ganpu tea by using ultra-high performance liquid chromatography (UHPLC)/quadrupole-time-of-flight tandem mass spectrometry (MS/MS) system. Xiao et al. (2021) identified 104 water-soluble compounds in Ganpu tea (made by Pu-erh raw tea and mandarin peel) by using UPLC-MS and UPLC-Q-TOF technologies, and found a remarkable decrease in the content of catechin and a remarkable increase in the content of alkaloids and flavonoids during the sun drying processing. Xu et al. (2021) identified 7 kinds of phenolic acids and 11 flavan-3-ols, as well as 27 flavonoids and flavonoid glycosides in pu-erh tea during solarized drying Ganpu tea by using UHPLC-Q-Orbitrap-MS/MS system. At present, most studies on quality formation of Ganpu tea are mainly centred on the sun drying process which is proved to be the metabolites significant changed stage (Xu et al., 2021, Xiao et al., 2021). However, sun drying mainly depends on the weather conditions, which is not conducive to the control and stability of tea quality. Furthermore, different processing technologies may result in distinct characteristic flavor. Until now, there are still limited studies on the flavor and quality formation of Ganpu tea by using different processing technologies and detection technologies.

Widely targeted metabolomics, with the advantages of nontargeted and targeted technologies, has been widely used in the detection of tea cultivars and processing (Wang et al., 2021, Zheng et al., 2021, Shi et al., 2021). However, due to the various kinds of raw materials for Ganpu tea processing, investigations concerning the chemical profile of Pu-erh during the process of Ganpu tea are still limited. In this study, *C. reticulata* Chachi pericarp from Xinhui county, Guangdong province of China and Pu-erh ripen tea from Menghai county, Yunnan province of China were used as materials for Ganpu tea production mainly by fixation and drying processing. This study was aimed to reveal the dynamic changes of non-volatile metabolites of Pu-erh during the process of Ganpu tea by using widely targeted metabolomic technology based on the ultra performance liquid chromatography–electrospray ionization-tandem mass spectrometry (UPLC–ESI-MS/MS) system, which can provide more information on the possible chemical basis for Pu-erh tea affected by Ganpu tea processing.

## Material and methods

### Tea samples

**Fig. 1:**
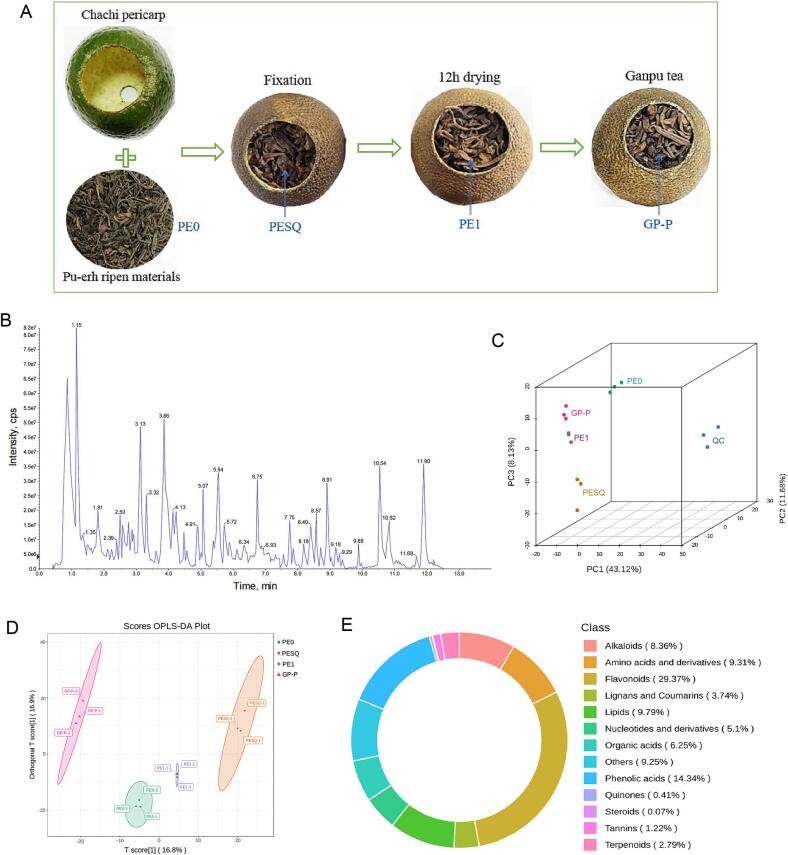
(A) The process of Ganpu tea. (B) Total ion flow (TIC) diagram of QC samples detected by mass spectrometry. (C) Three-dimensional PCA plot of non-volatiles in Pu-erh tea samples. (D) OPLS-DA of metabolic profiles. (E) The category of detected metabolites in Pu-erh tea samples.

Ganpu tea in this study was produced from Pu-erh ripen tea and *C. reticulata* Chachi pericarp. Ganpu tea and the materials were all provided by Dongguan Dayi Tea Co. Ltd. (Guangdong province, China). Pu-erh ripen tea was made by tea leaves of *Camellia sinensis* var. *assamica* produced from Menghai county, Yunnan province of China. The pericarp of Chachi was harvested from Xinhui county, Guangdong province of China. The process of Ganpu tea was shown in Fig. 1A. Briefly, a hole with the diameter of 2 cm was made at the top of the fresh Chachi fruit; the whole fresh peel of Chachi was separated from flesh and then filled with Pu-erh ripen tea; finally the encapsulated pericarp and Pu-erh tea were fixed at 80 ℃ for 2 h and then dried at 42℃ for 24 h. During the process of Ganpu tea, four Pu-erh tea samples were harvested to study the metabolites changes of Pu-erh tea, including raw Pu-erh ripen tea materials (PE0), Pu-erh tea after fixation (PESQ), 12 h dried Pu-erh tea sample (PE1) and 24 h dried Pu-erh tea (GP-P) (Fig. 1A). For each sample, 200 g of Pu-erh tea materials from Ganpu tea were mixed and prepared for further analysis.

### Sample preparation

The gathered tea samples were all freeze dried using a vacuum freeze-dryer (Scientz-100F, Zhejiang, China), then each freeze dried tea sample was milled by a mixer mill (MM 400, Retsch, Germany) with a zirconia bead for 90 s at 30 Hz. 50 mg of milled powder was dissolved with 1.2 mL of 70% methanol overnight at 4℃ (Merck, Germany). The extraction solution was vortexed for 30 s, centrifuged at 11,304*g* for 3 min, then the supernatants were absorbed and filtrated (ANPEL, Shanghai, China) before UPLC-MS/MS analysis. As the part of quality control process and system adjustment, quality control (QC) samples were made by blending equal volumes of the four tea samples. QC samples were performed in the same way with analytic tea samples. The Coefficient of Variation (CV) value of QC samples <0.3 (accounting for 95%) indicated the experimental data were very stable.

### UPLC conditions

The prepared tea samples were determined by UPLC–ESI-MS/MS system that was equipped with an Agilent SB-C18 column (1.8 µm, 2.1 mm × 100 mm, Foster City, CA). The UPLC conditions were as follows: mobile phase A, pure water (0.1% formic acid); mobile phase B, bacetonitrile (0.1% formic acid); column oven, 40℃; injection volume, 4 μL; flow velocity, 0.35 mL/min. The gradient programs referred to Wang et al. (2021).

### ESI-Q TRAP-MS/MS

The triple quadrupole (QQQ) and linear ion trap (LIT) scans acquired on UPLC-MS/MS system (API 4500 QTRAP) equipped with an ESI turbo ion-spray interface were conducted to the positive (+) and negative (-) ion mode by using Analyst 1.6.3 software (AB Sciex, Framingham, USA). The operation parameters of ESI source were conducted as follows: ion spray voltage (IS), 5500 V (+)/−4500 V (−); source temperature, 550 °C; ion source gas I (GSI), 50 psi; gas II (GSII), 60 psi; curtain gas (CUR), 25 psi. In the modes of LIT and QQQ, the mass calibration and instrument tuning were conducted with 100 and 10 μmol/L of polypropylene glycol solutions, respectively at Metware Biotechnology Co., Ltd. (Wuhan, China). QQQ scans were performed as multiple reaction monitoring (MRM), and the collision gas (nitrogen) was set as medium. By further optimization of the collision energy (CE) and declustering potential (DP), the individual MRM transitions of CE and DP was finished. A specific set of MRM transformations were monitored at every period based on the elution time of metabolites within this process. The metabolites were identified by comparing the retention time, fragmentation modes and the value of *m*/*z* to the standards in the databases (MetWare; MassBank, HMDB, and Metlin).

### Metabolites analysis

Principle component analysis (PCA) was an unsupervised multivariate statistical analysis method that was conducted to show the overview of the metabolomic analysis and overall differences between tea samples in each group and the variability degree between samples within the group in positive and negative mode. The significantly differential metabolites among tea samples were distinguished by orthogonal projections to latent structure-discriminant analysis (OPLS-DA). The hierarchical cluster analysis of the tea samples and identified metabolites in this study were shown in heatmaps with dendrograms, which was conducted by R package ComplexHeatmap. For hierarchical cluster analysis, a color spectrum was applied to show the normalized signal intensities of metabolites by unit variance scaling. Identified differential metabolites were annotated and then were mapped into corresponding biochemical pathways in the Kyoto Encyclopedia of Genes and Genomes (KEGG) compound database (https://www.kegg.jp/kegg/compound/). Heatmap analysis using TBtools (v1.082) (Chen et al., 2020) was applied to visualize the annotated metabolites in each sample. The annotated metabolites were then classified into the pathways that the metabolites were involved. Metabolic pathways were constructed based on pathway analysis of the differential metabolites detected in both positive and negative ion modes using MetaboAnalyst 3.0. The enrichment of the annotated metabolites in a pathway was indicated by P-value, and P ≤ 0.05 showed a significant enrichment. The k-means clustering analysis was applied to centralise and standardise the relative contents of the identified differential metabolites. Venn diagrams were conducted to show the differential metabolites between different tea groups.

### Statistical analysis

Differential metabolites analysis between two tea groups were selected by P-value (student’s *t* test), variable importance in project (VIP) and absolute Log_2_FC. Differential metabolites of multi-group analysis were selected by P-value (ANOVA) and VIP. Metabolites with significant differences in relative content were indicated by P < 0.05, VIP ≥ 1 and fold change ≥ 2 or ≤ 0.5. VIP values were calculated by OPLS-DA using R package MetaboAnalystR. Before OPLS-DA analysis, the data was log transform (log) and mean centered. To avoid overfitting, a permutation test (200 permutations) was conducted.

## Results and discussion

### The metabolites profiling of Pu-erh during Ganpu tea processing

**Supplementary Fig. S1:**
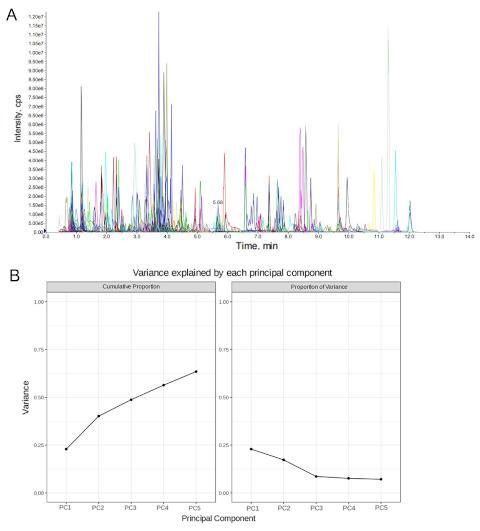


Both Pu-erh tea and citrus peel contain abundant phenolic acids, flavonoids, amino acids and other substances, which are responsible for their flavor and beneficial health properties (Lv et al., 2013, Yu et al., 2018). In this study, the popular Pu-erh ripen tea produced from Menghai county, Yunnan province of China and citrus peel planted in Xinhui county, Guangdong province of China were used to produce Ganpu tea, which were different with other studies that citrus tea were produced from dark tea “Tianjian” (Anhua county, Hunan province of China) by hot air drying (Qi et al., 2020) or Pu-erh raw tea by sun drying process (Xiao et al., 2021). Citrus tea possessing the characteristics of both citrus pericarp and tea endows tea product with unique flavor and multiple effects (Lv et al., 2013, Yu et al., 2018). To clarify the dynamic changes of non-volatiles in Pu-erh during the process of Ganpu tea and the possible chemical basis for Pu-erh tea affected by Ganpu tea processing, four Pu-erh tea samples (PE0, PESQ, PE1 and GP-P) were taken from the Ganpu tea during the processing as shown in Fig. 1A, and then were analyzed by using UPLC-MS/MS. As a part of the quality control process and system adjustment, QC samples mixed by equal volumes of all tea samples were performed in the same method with the analytic tea samples. The total ion flow (TIC) diagram of QC samples was overlaid to show the reliability of non-volatile metabolites detection (Fig. 1B). Each detected metabolite in this study was indicated in the diagram by a mass spectrum peak with different color (Fig. S1A). Both the TIC and multi-peak detection diagram in this study guaranteed the high stability of the instrument.

An unsupervised PCA was conducted to analyse the overview of the metabolites differences of the 15 samples in this study. In the model of the positive (or negative) mode, three biological replicates in each group clustered closely, and three QC samples were remarkably distinguished from the other four groups, which confirmed the reliability of the metabolites analysis (Fig. 1C). The PESQ group was below the QC group, while the other three groups PE0, PE1 and GP-P were almost above the QC group, indicating that the non-volatile metabolites in PESQ group are significantly different with that in other three groups. The principal components 1, 2 and 3 explained 43.12, 11.68 and 8.13% of the variation, respectively (Fig. 1C). The cumulative proportion of the PC1-PC5 reached 63.48%, and the proportion of variance for PC1, PC2, PC3, PC4 and PC5 were 22.89, 17.28, 8.58, 7.61 and 7.12%, respectively (Fig. S1B). Moreover, OPLS-DA analysis (R2X = 0.338, R2Y = 0.99, Q2 = 0.734) was performed to analyze the significantly different metabolites among the PE0, PESQ, PE1 and GP-P samples. The OPLS-DA score plot showed a clear separation among the four samples (Fig. 1D), which was in accordance with the PCA results. Tea are rich in a large number of secondary metabolites that are strongly associated with tea quality. In the four Pu-erh tea samples, a total of 1471 non-volatile metabolites were detected and identified, and the metabolites were divided into 13 classes including 432 kinds of flavonoids (accounting for 29.37% of the total metabolites), 211 phenolic acids (14.34%), 144 lipids (9.79%), 137 amino acids and derivatives (9.31%), 123 alkaloids (8.36%), 92 organic acids (6.25%), 75 nucleotides and derivatives (5.10%), 55 lignans and coumarins (3.74%), 41 terpenoids (2.79%), 18 tannins (1.22%), 6 quinones (0.41%), 1 steroids (0.07%) and 136 others (9.25%) (Fig. 1E). Tea and citrus peel have abundant flavonoids and phenolic acids, which are closely associated with the high antioxidant activity (Lv et al., 2013, Yu et al., 2018). Compared with the currently reported studies about the metabolites in citrus tea (Zheng et al., 2020, Xiao et al., 2021, Xu et al., 2021), more kinds of non-volatile metabolites had been detected in this study, which may be due to the different tea and citrus materials, as well as the different processing technologies and detection system.

### Dynamic changes of main differential metabolites during Ganpu tea processing

**Fig. 2:**
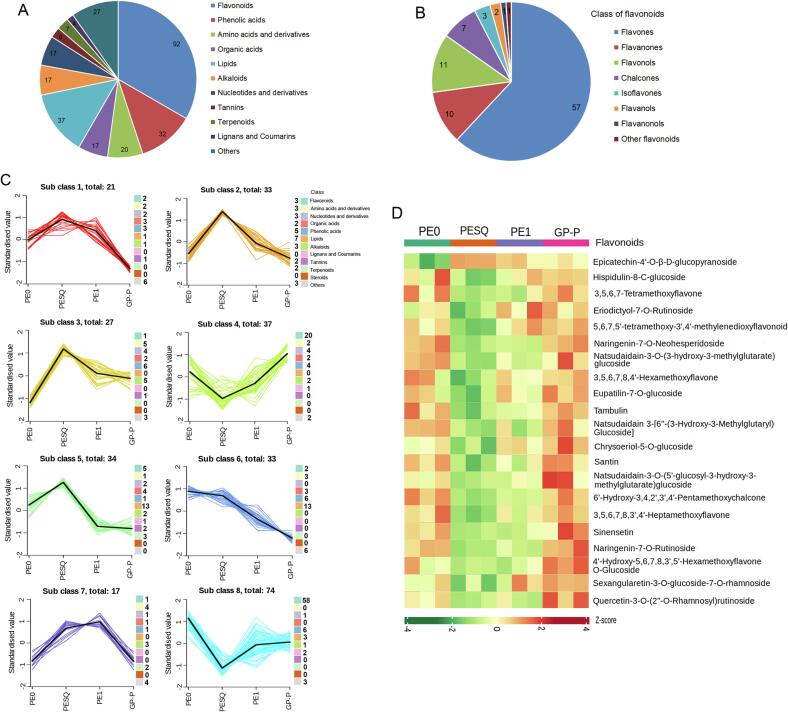
(A) The class of differential metabolites in Pu-erh during Ganpu tea processes. (B) Eight classes of differential metabolites in flavonoids. (C) K-means clustering analysis showing differential metabolites. (D) Heatmap of the relative content of the 21 flavonoids in subclass 3 and 4.

Among the 1471 non-volatile metabolites detected in the four Pu-erh tea samples, total 276 significantly differential metabolites among tea samples (PE0_vs_PESQ_vs_PE1_vs_GP-P) were screened according to the principle of P-value (<0.05) and VIP (>1). The identified differential metabolites in Pu-erh were divided into 11 classes including 92 kinds of flavonoids, 37 lipids, 32 phenolic acids, 20 amino acids and derivatives, 17 alkaloids, 17 organic acids, 17 nucleotides and derivatives, 7 terpenoids, 6 tannins, 4 lignans and coumarins, and 27 others (Fig. 2A). Quinones and steroids showed no significant changes during the processing. The 27 other metabolites mainly included saccharides and vitamins, as well as picrocrocin, jasminoside A, etc. Among the differential non-volatile metabolites in Pu-erh during Ganpu tea processing, the number of flavonoids were the most, mainly containing 57 kinds of flavones, 11 flavonols, 10 flavanones, 7 chalcones, 3 isoflavones, 2 flavanols, 1 flavanonols, etc. (Fig. 2B). Heatmap of the relative content of 92 flavonoids was shown in Fig. 2S. Different flavonoids have distinct physical, chemical, and physiological properties. The main flavonoids in Pu-erh were kaempferol, myricetin, quercetin, and their glycosides (Xu et al., 2021). Flavonols are mainly present in the forms of glycosides including C-glycosides and *O*-glycosides in tea, and there are three major types of flavonols including kaempferol, quercetin and myricetin (He et al., 2021). In this study, flavonols present in the form of *O*-glycosides were the main differential metabolites, such as kaempferol-3-*O*-sulfonate, myricetin-3-*O*-sulfonate, quercetin-3-*O*-galactoside, etc (Fig. 2S). The major flavanols in Pu-erh were catechin/gallocatechin and its dimers or gallate derivatives, however, the content of catechins showed no significant changes (P > 0.05, VIP < 1) in Pu-erh during the Ganpu tea processing in this study. Flavones and catechins were the main compounds which contributed to the health benefits of Pu-erh tea and affected the bitter and astringent taste of tea infusion (Zhu et al., 2017). Catechins can be converted to thearubigins, puerins A and B, oligomers and other polymeric compounds in dark tea during the post-fermentation of microorganisms, resulting in the relatively low content of catechins and high level of theaflagallin and epiafzelechin (Luo et al., 2012, Zhu et al., 2016).

To analyse the change trend of significant differential metabolites in Pu-erh during Ganpu tea processing, the relative contents of differential metabolites were centralized and standardized, and then were clustered into 8 subclasses based on the variation pattern of metabolites by K-means analysis. As shown in Fig. 2C, among the 8 subclasses, the differential metabolites in subclass 8 were the most enriched in the four samples, followed by subclass 4. In both subclass 8 and 4, flavonoids were the main differential metabolites. The content of 111 non-volatiles in subclass 4 and 8 were decreased from PE0 to PESQ and then were increased from PESQ to GP-P, while the content of 115 non-volatile metabolites in subclass 1, 2, 3 and 5 were increased from PE0 to PESQ and then were decreased from PESQ to GP-P during the Ganpu tea processing (Fig. 2C). However, in subclass 3 and 4, the relative content of non-volatiles in GP-P were obviously higher than that in PE0. Among the metabolites, the number of flavonoids was the most, such as epicatechin-4′-*O*-β-d-glucopyranoside, eupatilin-7-*O*-glucoside, quercetin-3-*O*-(2″-*O*-rhamnosyl) rutinoside, naringenin-7-*O*-neohesperidoside, chrysoeriol-5-*O*-glucoside, naringenin-7-*O*-rutinoside, natsudaidain-3-*O*-(5′-glucosyl-3-hydroxy-3-methylglutarate) glucoside, sinensetin, etc. (Fig. 2D). Heatmap of the relative content of the 21 flavonoids in subclass 3 and 4 was shown in Fig. 2D. Furthermore, the content of 17 non-volatiles in subclass 7 were increased from PE0 to PE1 and then were decreased from PE1 to GP-P, which showed similar accumulation levels between raw Pu-erh tea materials PE0 and GP-P (Fig. 2C). The relative content of 33 non-volatile metabolites in subclass 6 continued to decrease during Ganpu tea processing, and 39.39% of decreased metabolites were lipids that included free fatty acids (hydroxy ricinoleic acid, eicosadienoic acid, 11-octadecanoic acid, etc.) and LPC (lysoPC 15:1, lysoPC 16:1, lysoPC 14:0, lysoPC 18:2, etc.) (Fig. 2C and Fig. S3). Among the 37 significant differential lipids, most of the lipids in GP-P were significantly decreased compared with that in PE0, and the relative content of lipids were visualized in heatmap as shown in Fig. S3.

### Changes of the main differential non-volatile metabolites during Ganpu tea processing

**Fig. 3:**
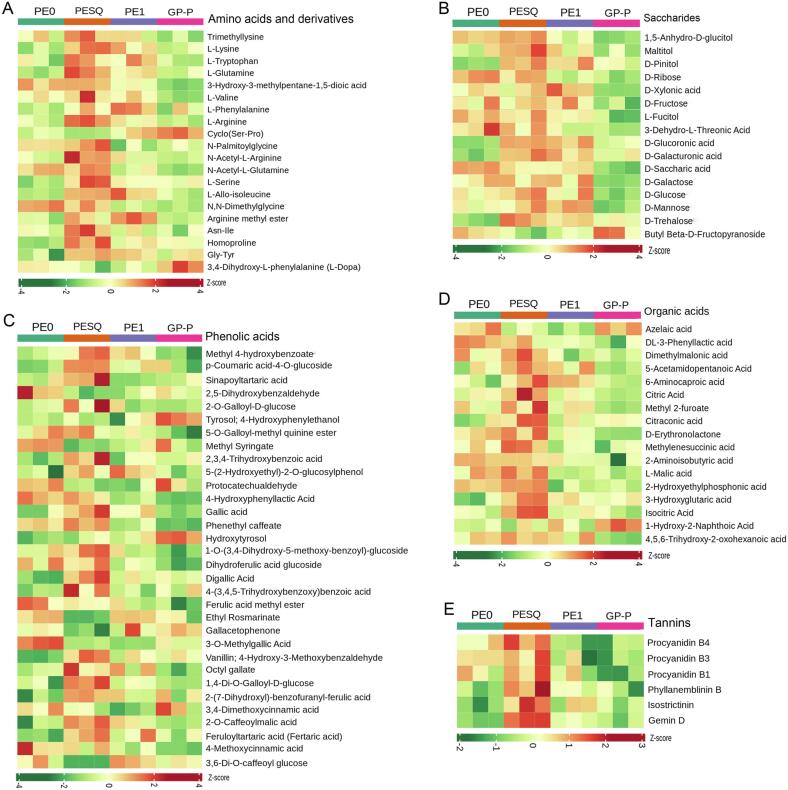
Heatmap of the relative content of the differential non-volatiles in Pu-erh during Ganpu tea processing. (A) Amino acids and their derivatives. (B) Saccharides. (C) Phenolic acids. (D) Organic acids. (E) Tannins.

Tea are rich in a large number of secondary metabolites, such as tea polyphenols, phenolic acids, free amino acids, alkaloids, organic acids and tannins, which are strongly associated with tea quality and form the rich taste and unique flavour of tea (Farag et al., 2023, Wei et al., 2018, Zhu et al., 2016, Zheng et al., 2021). Heatmap analysis was applied to visualize the variation of main quality related metabolites in Pu-erh during Ganpu tea processing (Fig. 3). Generally, free amino acids, especially theanine, are proved to be associated with the umami taste of tea infusion (Kimutai et al., 2016), and the intensity of taste increases with the concentration of amino acids (Narukawa et al., 2008). The content of free amino acids in dark tea is relatively low due to the Maillard reactions and microorganisms which can convert some amino acids to volatile aldehydes during the post-fermentation process (Luo et al., 2012, Zhu et al., 2016). However, different amino acids showed different changes during Ganpu tea processing in this study. Total 20 differential amino acids and derivatives were detected in Pu-erh (Fig. 3A). As shown in Fig. 3A, most of the amino acids and derivatives such as trimethyllysine, l-lysine, l-tryptophan, l-glutamine, 3-hydroxy-3-methylpentane-1,5-dioic acid, l-valine, l-arginine, l-serine, *N*-palmitoylglycine, *N*-acetyl-l-arginine, *N*-acetyl-l-glutamine, l-allo-isoleucine, Asn-Ile, Gly-Tyr, and homoproline were significantly increased in PESQ, and then were decreased to different levels in low temperature drying process, suggesting that high temperature may promote the hydrolysis of protein during the process of fixation and the amino acid formation rate is higher than the consumption (Wan, 2003). The relative content of l-phenylalanine and arginine methyl ester were significantly increased in PESQ and PE1, but were decreased in GP-P, while the relative content of N, *N*-dimethylglycine continued to decrease during the processing (Fig. 3A). Compared with PE0, the levels of l-phenylalanine, cyclo (Ser-Pro), l-serine, homoproline, Gly-Tyr and 3,4-dihydroxy-l-phenylalanine (l-dopa) were significantly increased in GP-P (Fig. 3A). Phenylalanine is reported to have umami taste or enhance umami and sweet tastes by collaboration with other amino acids (Procopio et al., 2013). Theanine, as the major amino acid in tea, accounts for 50–80% of the total free amino acids in tea (Tan et al., 2017, Kimutai et al., 2016), and during the processing of Ganpu tea, the content of theanine in Pu-erh showed no significant different (P > 0.05, VIP < 1). The change trend of free amino acids (12.58–14.83 mg/g) and theanine (10.33–12.43 mg/g) in Pu-erh during Ganpu tea processing were similar, which slightly increased in PESQ and then continued to decrease in drying process (Fig. S4).

Soluble sugar is the main water-soluble carbohydrate in tea, which contributes significantly to sweet taste of tea. In this study, the content of soluble sugar (25.8–27.9 mg/g) in Pu-erh during Ganpu tea processing were similar showing no significant difference (P > 0.05) (Fig. S4B). However, the composition of sugars were significantly different, and total 16 differential sugars were detected (P < 0.05, VIP > 1), including the most common monosaccharides and disaccharides. Sucrose, fructose and glucose are the major sugars both in citrus and tea (Albertini et al., 2006). The sweetness of tea depends not only on the levels of various sugar substances but also on the proportions of various components. As shown in Fig. 3B, most of the sugars including 1,5-anhydro-d-glucitol, maltitol, d-pinitol, d-glucoronic acid, d-galacturonic acid, d-glucose, d-galactose, d-trehalose and d-mannose were found to significantly accumulate in PESQ after fixation, suggesting that high temperature can promote the formation of these soluble sugars. However, after the low temperature drying process, the levels of these sugars were significantly decreased in GP-P. Compared with PE0, only 5 differential sugars such as d-pinitol, d-glucoronic acid, d-galacturonic acid, d-trehalose and butyl beta-d-fructopyranoside were significantly increased in GP-P (Fig. 3B), indicating that the accumulation of most sugars in Pu-erh were negatively affected by the processing.

Phenolic acids are important metabolites in tea which contribute to the taste and color of tea infusion (Dai et al., 2015). Total 32 kinds of differential phenolic acid substances including benzoic acid congeners and cinnamic acid congeners were detected (VIP > 1, P < 0.05), and most of the phenolic acids such as gallic acid, methyl 4-hydroxybenzoate, p-coumaric acid-4-*O*-glucoside, sinapoyltartaric acid, 2,3,4-trihydroxybenzoic acid, digallic acid, etc., were significantly increased after fixation (Fig. 3C). Gallic acid has been proved to contribute to the umami taste of tea, and is a precursor of methyl gallate and catechins (Zhou et al., 2020). After fixation, the content of gallic acid continued to decrease during drying process (Fig. 3C). Compared with PE0, the gallic acid content in GP-P were significantly decreased. Xu et al. (2021) reported that the content of gallic acid was significantly decreased after co-fermented with citrus peel, which was consistent with our results. In contrast, the levels of 2,5-dihydroxybenzaldehyde, methyl syringate, 4-hydroxyphenyllactic acid, ferulic acid methyl ester, 3-*O*-methylgallic acid and 4-methoxycinnamic acid were down regulated during the Ganpu tea processing, suggesting that these metabolites were significantly influenced by the heat of fixation and drying (Fig. 3C), which was consistent with previous studies that strong hydrolysis and redox reactions during fixation could promote the transformation of phenolic acid substances (Dai et al., 2020).

Organic acids are closely associated with the sour and fruity taste of green tea, and are also the key intermediate products involving in carbohydrate catabolism (Deng & Ashihara, 2015). A total of 17 organic acid substances were detected (P < 0.05, VIP > 1), and most of the organic acids were significantly increased in PESQ after fixation, but then continued to reduce during drying process, such as dimethylmalonic acid, 5-acetamidopentanoic acid, 6-aminocaproic acid, citric acid, methyl 2-furoate, citraconic acid, d-erythronolactone, methylenesuccinic acid, l-malic acid, 2-hydroxyethylphosphonic acid, 3-hydroxyglutaric acid, isocitric acid and 4,5,6-trihydroxy-2-oxohexanoic acid (Fig. 3D). On the contrary, 1-hydroxy-2-naphthoicacid, DL-3-phenyllactic acid and 2-aminoisobutyric acid continued to increase during Ganpu tea processing, suggesting that the heat of fixation and drying promote the formation of these metabolites (Fig. 3D). During the green tea processing, the contents of anchoic acid, citric acid and quinic acid were reported to show significant increases (Wang et al., 2021). Among the significantly differential metabolites in Pu-erh during Ganpu tea processing, citric acid, methyl 2-furoate and isocitric acid were the most significant organic acids (VIP > 2) (Fig. 3D).

Tannins are important polyphenols in tea which may contribute to the astringent taste of tea (Tan et al., 2017). Six differential tannins including procyanidin B1, procyanidin B3, procyanidin B4, phyllanemblinin B, isostrictinin and gemin D were detected during Ganpu tea processing, which showed similar change trend that were all significantly accumulated in PESQ after fixation and then were significantly decreased during the drying process (Fig. 3E). Procyanidins belong to flavonoids, and procyanidins structures are easily affected by many factors including acidity, temperature, light, alkalinity, etc. (Luo et al., 2013). In general, fixation was the key procedure of Ganpu tea processing in which most of the main tea taste related metabolites such as flavonoids, amino acids, phenolic acids, soluble sugars, organic acids and tannins were remarkably decreased in Pu-erh after fixation (Fig. 3). The unique flavor of Ganpu tea was the comprehensive action of these substances, which was significantly influenced by the processing technologies.

### Differential metabolites profile of Pu-erh in different Ganpu tea processes

**Fig. 4:**
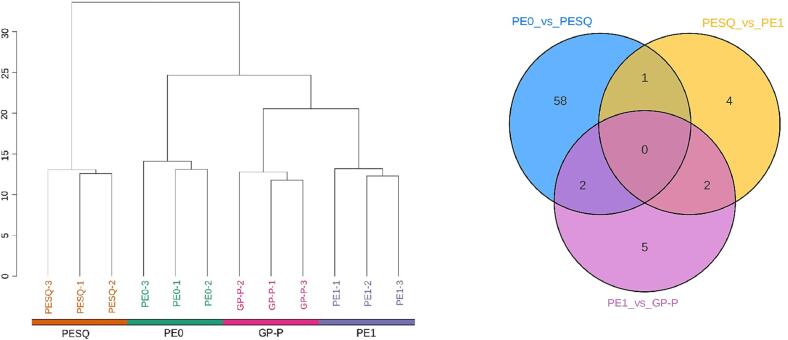
(A) Hierarchical cluster analysis of the similarity among tea samples. (B) Venn diagram showing the overlapping relationship of differential metabolites of PE0_vs_PESQ, PESQ_vs_PE1 and PE1_vs_GP-P.

To reveal the influence of each process on the non-volatiles of Pu-erh present at the different stages of Ganpu tea processing, the significantly differential accumulated metabolites between the processes were investigated. As shown in Fig. 4A, hierarchical cluster analysis based on different tea samples was conducted to show the similarity among samples, and the results showed that PE1 and GP-P clustered in the same cluster had the highest similarity, followed in order by PE0. Furthermore, PESQ was clustered into a single branch, indicating that PESQ had the greatest difference from other samples (Fig. 4A). OPLS-DA was performed to distinguish the significantly differential metabolites of PE0_vs_PESQ, PESQ_vs_PE1 and PE1_vs_GP-P according to the principle of P < 0.05, VIP ≥ 1 and fold change ≥2 or ≤0.5 (Fig. S4). A Venn plot was constructed based on the differential metabolites from PE0_vs_PESQ, PESQ_vs_PE1 and PE1_vs_GP-P, and a total of 72 differential metabolites were identified between any two stages (Fig. 4B). Among the three groups, the most differential metabolites were detected in PE0_vs_PESQ, further confirming that fixation was the key step of Ganpu tea processing, which was consistent with the hierarchical cluster analysis. Total 61, 7 and 9 differential metabolites were detected in PE0_vs_PESQ, PESQ_vs_PE1 and PE1_vs_GP-P, respectively. A total of 58 differential metabolites were unique to PE0_vs_PESQ, which were only significantly affected by the fixation of Ganpu tea processing (Fig. 4B). Furthermore, only 1 differential metabolite (dihydroxy-dimethoxyflavone) was common between PE0_vs_PESQ and PESQ_vs_PE1, 2 common metabolites (troxerutin and lysoPE 18:0 (2n isomer)) were found between PESQ_vs_PE1 and PE1_vs_GP-P, and PE0_vs_PESQ and PE1_vs_GP-P also possessed 2 common metabolites including 5′-deoxy-5′-(methylthio) adenosine and quercetin-3-*O*-(2′'-*O*-rhamnosyl) rutinoside (Fig. 4B), indicating that the common metabolites were simultaneously affected by the fixation and low temperature drying processes.

### Significantly differential accumulated metabolites in fixation process

**Fig. 5:**
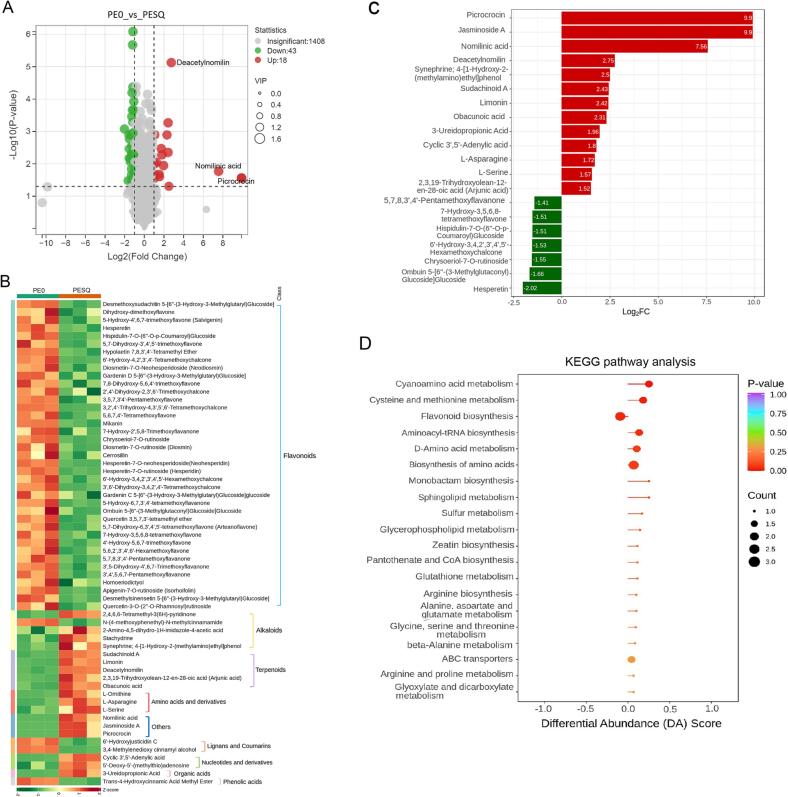
(A) A volcano plot showing the differential non-volatiles between PE0 and PESQ. (B) Heatmap of the relative content of the differential non-volatile metabolites in PE0 and PESQ. (C) A bar chart showing the top 20 significantly differential metabolites. (D) KEGG analysis of the annotated differential metabolites in metabolic pathway.

To further clarify the significantly differential metabolites between PE0 and PESQ after fixation, a volcano plot was conducted. As shown in Fig. 5A, compared with PE0, 18 metabolites were significantly increased in PESQ after fixation, while 43 were decreased (P < 0.05, VIP ≥ 1 and fold change ≥2 or ≤0.5). Among the 61 differential metabolites, 39 were flavonoids, accounting for 63.9% of all these metabolites, and the other metabolites included 5 alkaloids, 5 terpenoids, 3 amino acids and derivatives, 2 lignans and coumarins, 2 nucleotides and derivatives, 1 organic acids, 1 phenolic acids and 3 others (Fig. 5B and Table S1). Both tea and citrus pericarp are rich in flavonoids (Xu et al., 2021, Jeong et al., 2022). However, 39 flavonoids as well as 2 lignans and coumarins (6′-hydroxyjusticidin C and 3,4-methylenedioxy cinnamyl alcohol), were all significantly decreased after fixation (Fig. 5B). Xiao et al. (2021) found a significant increase in flavonoid contents during the sun drying processing of Ganpu tea, which was inconsistent with our results. This may be due to the different processing technologies and materials. In the study of Xiao et al. (2021), Pu-erh raw tea and mandarin peel were used as materials for Ganpu tea processing, and the flavor characteristics of Ganpu tea were studied. On the contrary, 5 terpenoids (i.e., limonin, obacunoic acid, sudachinoid A), 3 amino acids (l-ornithine, l-asparagine and l-serine), 1 organic acids (3-ureidopropionic acid), and 2 nucleotides and derivatives (5′-deoxy-5′-(methylthio) adenosine and cyclic 3′,5′-adenylic acid) were all significantly increased in this study (Fig. 5B). The three other differential metabolites such as jasminoside A (Log_2_FC = 9.90), picrocrocin (Log_2_FC = 9.90) and nomilinic acid (Log_2_FC = 7.56) were newly detected and also significantly increased after fixation (Fig. 5B). A bar chart was conducted to show the top 20 significantly differential metabolites in PE0_vs_PESQ according to the fold change, as shown in Fig. 5C. Red indicates an increase in relative content of a metabolite, while green indicates a decrease in relative content of a metabolite. Among the 20 metabolites, 13 (i.e., jasminoside A, picrocrocin, nomilinic acid, limonin) were up regulated, while 7 (i.e., hesperetin, ombuin 5-[6′'-(3-methylglutaconyl) glucoside] glucoside, chrysoeriol-7-*O*-rutinoside) were down regulated. Jasminoside A is a new bisdesmoside isolated from the methanolic extract of *Solanum jasminoides* (Ohno et al., 2012), and picrocrocin is a main component with high amount in *Crocus sativus* (Yue et al., 2020). Both jasminoside A and picrocrocin were barely detected in tea, indicating that these compounds were formed during Ganpu tea processing. Nomilinic acid and limoninis belonging to the family of limonoid mainly exist in the family of *Rutaceae* including oranges, grapefruits, lemons, and other fruits, which are closely associated with the bitter taste in citrus fruits and mainly consumed for medicine and food (Bennett et al., 1989). Therefore, the increased limonoid in Pu-erh tea may be from the pericarp of *C. reticulata* Chachi. On the other hand, kaempferol, quercetin, rutin, hesperidin and hesperetin are detected in orange peel and tea, and both hesperidin and hesperetin have important biological properties such as antioxidant potential, anti-inflammatory, anticancer and antimicrobial effects (Samadi and Fard, 2020, Ortiz et al., 2022). Hesperetin, hesperetin-7-*O*-neohesperidoside (neohesperidin), hesperetin-7-*O*-rutinoside (hesperidin), and quercetin-3-*O*-(2′'-*O*-rhamnosyl) rutinoside detected in this study were found to show a downward trend after fixation (Fig. 5B and C), which was inconsistent with a previous study (Xu et al., 2021), in which most of the quercetin glycosides were significantly increased, and hesperidin was found in Pu-erh tea after co-fermented with citrus.

The differential metabolites between PE0 and PESQ were annotated based on KEGG database, which were mainly enriched in flavonoid biosynthesis, biosynthesis of amino acids, sulfur metabolism, cysteine and methionine metabolism, cyanoamino acid metabolism, aminoacyl-tRNA biosynthesis, d-amino acid metabolism, glutathione metabolism, monobactam biosynthesis, etc. (Fig. 5D). As shown in Fig. 5D, KEGG differential abundance (DA) score was applied to reflect the overall variation of all metabolites in metabolic pathways. A score of 1 indicated the change trend of all identified metabolites in the pathway was up-regulated, and a score of −1 indicated the change trend was down-regulated. The length of the line segment represented the absolute value of the differential abundance score. The size of the dot represented the number of differential metabolites in the metabolic pathway. The color of line segments and dots reflected the size of P-value that was shown in purple (large) to red (small) scale. In this study, the metabolites involved in the metabolic pathways of flavonoid biosynthesis and biosynthesis of amino acids were more abundant and showed significant changes (Fig. 5D). Dots distributed on the left side of the central axis indicated that the overall variation of metabolites in the pathway tended to be down-regulated, while dots distributed on the right indicated that the overall variation of metabolites in the pathway tended to be up-regulated. Therefore, the differential metabolites in flavonoid biosynthesis showed a downward trend, while the differential metabolites in biosynthesis of amino acids showed an upward trend (Fig. 5D). l-ornithine, l-asparagine and l-serine were the most significantly increased amino acids and derivatives. l-serine was proved to contribute to the sweet taste (Kirimura et al., 1969). As the most important bioactive components of citrus, flavonoids have a relatively high abundance in citrus peels and the biosynthesis pathway of flavonoids occurs through the general phenylpropanoid pathway (Li and Schluesener, 2017, Zhao et al., 2020). However, most of the flavonoids in Pu-erh tea during Ganpu tea processing showed a downward trend, suggesting that flavonoids can be easily affected by heat or temperature (Luo et al., 2013). High temperature can promote the hydrolysis, oxidation, isomerization and other thermochemical reactions of the metabolites.

### Significantly differential accumulated metabolites in drying process

**Fig. 6:**
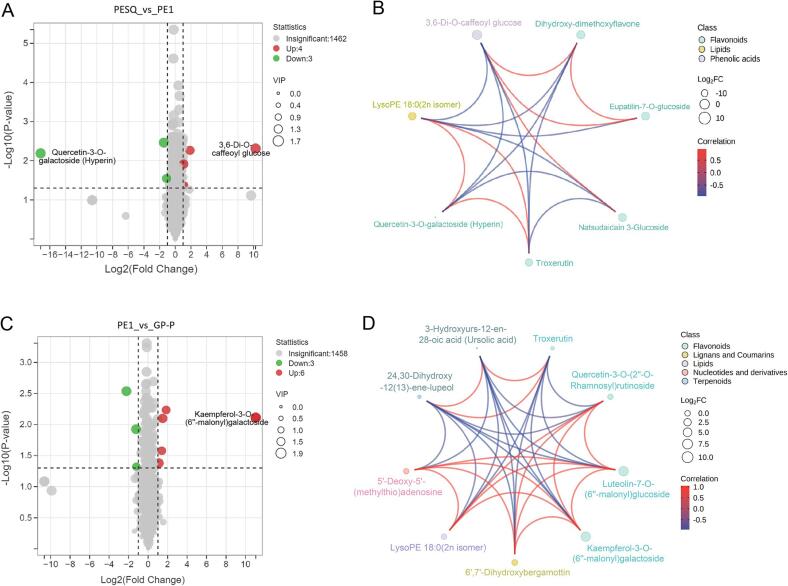
(A) A volcano plot showing the differential non-volatile metabolites between PESQ and PE1. (B) Chord diagram showing the differential metabolites of PESQ_vs_PE1. (C) A volcano plot showing the differential non-volatile metabolites between PE1 and GP-P. (D) Chord diagram showing the differential metabolites of PE1_vs_GP-P. Different colors in the figure represent different classes of metabolites; the line represents the Pearson correlation coefficient between the corresponding differential metabolites, the red line represents positive correlation, and the blue line represents negative correlation. (For interpretation of the references to color in this figure legend, the reader is referred to the web version of this article.)

Low temperature drying is an important and last procedure for the quality formation of Ganpu tea. So far, most studies on Ganpu tea are centred on sun drying process (Xu et al., 2021, Xiao et al., 2021), and there are limited information on the low temperature drying process. Generally, the drying of Ganpu tea needs 24 h in this study, and 12 h dried Pu-erh (PE1) and 24 h dried Pu-erh (GP-P) tea samples were harvested. GP-P was from the final Ganpu tea product. By comparison, the sun drying processing needs almost 15 d of high humidity and high temperature conditions or 4 months in the circumstance of 40 ℃ and 75% humidity (Xu et al., 2021), causing flavor related compounds of Ganpu tea to be remarkably changed. To clarify the significantly differential metabolites of Pu-erh during drying process of Ganpu tea, volcano plots of PESQ_vs_PE1 and PE1_vs_GP-P were conducted. As shown in Fig. 6A, compared with PESQ, 4 metabolites were significantly increased in PE1 after 12 h drying, while 3 were decreased (P < 0.05, VIP ≥ 1 and fold change ≥2 or ≤0.5). Among the 7 differential metabolites, 5 were flavonoids, accounting for 71.4% of all these metabolites, and the other metabolites were 1 lipids and 1 phenolic acids. Three flavones based on the backbone of 2-phenylchromen-4-one (Zhao et al., 2020, Zheng et al., 2018, He et al., 2021) including dihydroxy-dimethoxyflavone, eupatilin-7-*O*-glucoside and natsudaidain 3-glucoside, and 1 phenolic acids (3,6-di-*O*-caffeoyl glucose) were significantly increased (Fig. 6B). On the contrary, three metabolites such as quercetin-3-*O*-galactoside (flavonols), lysoPE 18:0 (2n isomer) (lipids) and troxerutin (flavones) were significantly decreased (Fig. 6B). There is synergistic or mutually exclusive relationship between different metabolites. In this study, correlation analysis based on Pearson correlation coefficient was used to analyse the metabolic proximities between significantly different metabolites, which helps to further understand the mutual regulatory relationship between metabolites during the process of Ganpu tea. The red line in the diagram indicated positive correlation, and the blue line represented negative correlation. As shown in Fig. 6B, the three down regulated metabolites quercetin-3-*O*-galactoside, lysoPE 18:0 (2n isomer) and troxerutin showed positive correlation, and 3 of the 4 up regulated metabolites (dihydroxy-dimethoxyflavone, eupatilin-7-*O*-glucoside and 3,6-di-*O*-caffeoyl glucose) also showed positive correlation. Furthermore, natsudaidain 3-glucoside was only positively correlated with 3,6-di-*O*-caffeoyl glucose. In contrast, the 4 up regulated metabolites were negatively correlated with the 3 down regulated metabolites (Fig. 6B).

**Supplementary Fig. S2:**
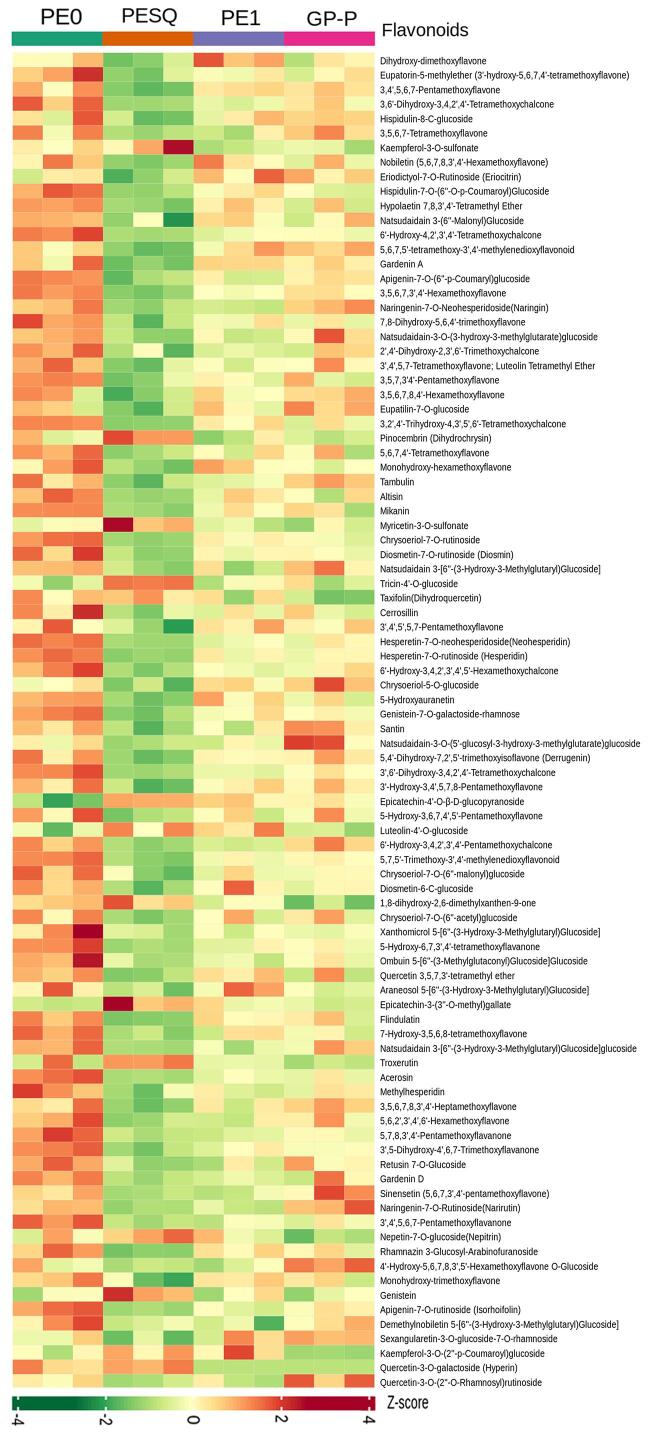


**Supplementary Fig. S3:**
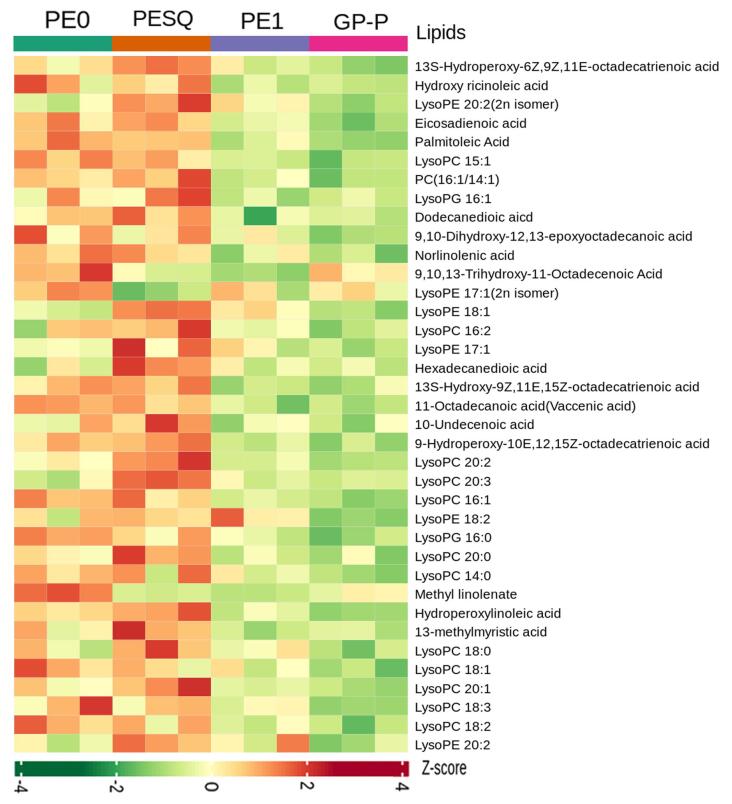


**Supplementary Fig. S4:**
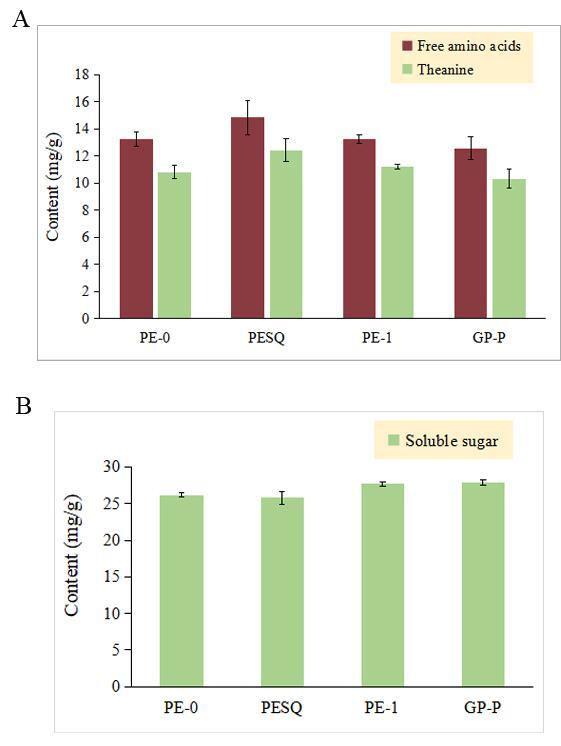


Next, significantly differential metabolites between PE1 and GP-P were analyzed, which were shown in a volcano plot of PE1_vs_GP-P (Fig. 6C). Total 9 significantly differential metabolites were detected, including 4 kinds of flavonoids, 1 lipids, 1 lignans and coumarins, 1 nucleotides and derivatives and 2 terpenoids (P < 0.05, VIP ≥ 1 and fold change ≥2 or ≤0.5). Compared with PE1, 6 metabolites were significantly increased in GP-P after 24 h drying, while 3 were decreased (Fig. 6C). The three down regulated metabolites such as troxerutin (flavones), 24,30-dihydroxy-12(13)-ene-lupeol (triterpene) and 3-hydroxyurs-12-en-28-oic acid (triterpene) showed positive correlation with each other (Fig. 6D). Troxerutin was the common metabolite that was significantly decreased both in PESQ_vs_PE1 and PE1_vs_GP-P during drying process, indicating that drying process negatively affected the accumulation of troxerutin (Fig. 6B and D), however, the level of troxerutin was remarkably accumulated in PESQ (Fig. S2). Troxerutin (vitamin P4) is a derivative of naturally occurring bioflavonoid rutin reported in tea, cereal grains, coffee and vegetables, which has various biological activities including anti-inflammatory, antioxidant, etc. (Ahmadi et al., 2021). However, the three down regulated metabolites were negatively correlated with the 6 up regulated metabolites (Fig. 6D). Furthermore, the 6 up regulated metabolites including 5′-deoxy-5′-(methylthio) adenosine (nucleotides and derivatives), lysoPE 18:0 (2n isomer) (lipids), 6′,7′-dihydroxybergamottin (coumarins), quercetin-3-*O*-(2′'-*O*-rhamnosyl) rutinoside (flavonols), luteolin-7-*O*-(6′'-malonyl) glucoside (flavones) and kaempferol-3-*O*-(6′'-malonyl) galactoside (flavonols) were positively correlated with each other (Fig. 6D). Results of this study fully revealed the effects of high temperature fixation and lower drying process on the non-volatile metabolites of Pu-erh during Ganpu tea processing. Food dried by heat is usually thermally affected by high temperatures and oxygen, hence the dehydrated foods is generally brown in color. However, freeze-drying performed under a vacuum at low temperature can cause less changes in color, texture and flavor of citrus peels than other drying method (Jeong et al., 2022). Ganpu tea made by different raw materials and processing technologies may have unique flavor, thus more studies on the quality formation of Ganpu tea are still underway.

## Conclusion

Ganpu tea is a novel tea product with pleasant and unique flavor that has both the characteristics of tea and citrus peel. In this study, total 276 significantly differential metabolites in Pu-erh during Ganpu tea processing were identified, 92 flavonoids (accounting for 33.3% of the total differential metabolites) were the main differential metabolites, and the change trend of differential metabolites were clustered into 8 subclasses based on the variation pattern of metabolites by K-means analysis. The influence of each process on the non-volatiles of Pu-erh present at the different stages of Ganpu tea processing revealed that total 72 differential metabolites were identified between any two stages and fixation was the key step with 61 differential metabolites. Among the 61 metabolites, 39 flavonoids (i.e., hesperetin, ombuin 5-[6′'-(3-Methylglutaconyl)glucoside]glucoside, diosmetin-7-*O*-rutinoside), and 2 lignans and coumarins were significantly decreased, while 5 terpenoids (i.e., deacetylnomilin, limonin, sudachinoid A), 3 amino acids (i.e., l-serine, l-asparagine), 2 nucleotides and derivatives, and 1 organic acids were significantly increased. 3 newly detected metabolites such as jasminoside A (Log_2_FC = 9.90), picrocrocin (Log_2_FC = 9.90) and nomilinic acid (Log_2_FC = 7.56) were most significantly increased after fixation. Results of this study fully revealed the dynamic changes of non-volatiles in Pu-erh during Ganpu tea processing. At present, a variety of Ganpu teas that made by pericarp of different citrus cultivars at different maturity and various kinds of tea materials are available in the tea market, hence more studies are still needed.

## CRediT authorship contribution statement

**Xinyi Deng:** Investigation, Data curation, Methodology, Writing – original draft. **Shiqiang He:** Investigation, Methodology, Data curation. **Yuxin Han:** Formal analysis, Methodology. **Yingjuan Chen:** Conceptualization, Methodology, Data curation, Supervision, Writing – review & editing.

## Declaration of Competing Interest

The authors declare that they have no known competing financial interests or personal relationships that could have appeared to influence the work reported in this paper.

## Data Availability

Data will be made available on request.
