# Supplementary material for: Metabolites profiling reveals the dynamic changes of non-volatiles in Pu-erh during Ganpu tea processing
Source: Food Chem X. 2023 Jun 26;19:100774. doi: 10.1016/j.fochx.2023.100774 (PMC10534103; doi:10.1016/j.fochx.2023.100774)
Supplement: Supplementary data 1 [file mmc1.docx]

**Table S1** The retention time, the precursor ion, the production and collision energy of 61 significantly differential metabolites in fixation process.

| Compounds | Class I | Retention time | Precursor ion | Collision energy |
| --- | --- | --- | --- | --- |
| Desmethoxysudachitin 5-[6''-(3-Hydroxy-3-Methylglutaryl)Glucoside] | Flavonoids | 4.70 | 637.18 | 30 |
| 2,4,6,6-Tetramethyl-3(6H)-pyridinone | Alkaloids | 2.30 | 152.11 | 30 |
| Dihydroxy-dimethoxyflavone | Flavonoids | 6.60 | 315.09 | 30 |
| 5-Hydroxy-4',6,7-trimethoxyflavone | Flavonoids | 5.40 | 329.10 | 30 |
| Hesperetin | Flavonoids | 5.90 | 303.09 | 30 |
| Hispidulin-7-O-(6''-O-p-Coumaroyl)Glucoside | Flavonoids | 4.20 | 609.18 | 30 |
| 5,7-Dihydroxy-3',4',5'-trimethoxyflavone | Flavonoids | 6.90 | 345.10 | 40 |
| Hypolaetin 7,8,3',4'-Tetramethyl Ether | Flavonoids | 6.00 | 359.11 | 30 |
| 6'-Hydroxy-4,2',3',4'-Tetramethoxychalcone | Flavonoids | 7.30 | 345.13 | 30 |
| L-Ornithine | Amino acids and derivatives | 0.70 | 133.10 | 20 |
| Diosmetin-7-O-Neohesperidoside | Flavonoids | 4.10 | 609.18 | 20 |
| L-Asparagine | Amino acids and derivatives | 0.70 | 133.06 | 20 |
| Gardenin D 5-[6''-(3-Hydroxy-3-Methylglutaryl)Glucoside] | Flavonoids | 5.50 | 681.20 | 30 |
| 7,8-Dihydroxy-5,6,4'-trimethoxyflavone | Flavonoids | 6.80 | 345.10 | 30 |
| N-(4-methoxyphenethyl)-N-methylcinnamamide | Alkaloids | 7.50 | 296.16 | 30 |
| Cyclic 3',5'-Adenylic acid | Nucleotides and derivatives | 1.80 | 328.05 | -20 |
| 2',4'-Dihydroxy-2,3',6'-Trimethoxychalcone | Flavonoids | 7.80 | 331.12 | 30 |
| 2-Amino-4,5-dihydro-1H-imidazole-4-acetic acid | Alkaloids | 0.90 | 144.08 | 30 |
| 3,5,7,3'4'-Pentamethoxyflavone | Flavonoids | 6.60 | 373.13 | 30 |
| 3,2',4'-Trihydroxy-4,3',5',6'-Tetramethoxychalcone | Flavonoids | 6.80 | 377.12 | 30 |
| 5,6,7,4'-Tetramethoxyflavone | Flavonoids | 6.60 | 343.12 | 30 |
| 3-Ureidopropionic Acid | Organic acids | 0.80 | 131.05 | -30 |
| Sudachinoid A | Terpenoids | 6.10 | 489.22 | -30 |
| Mikanin | Flavonoids | 6.60 | 345.10 | 30 |
| 7-Hydroxy-2',5,8-Trimethoxyflavanone | Flavonoids | 7.80 | 331.12 | 30 |
| Chrysoeriol-7-O-rutinoside | Flavonoids | 4.00 | 607.17 | -30 |
| Diosmetin-7-O-rutinoside | Flavonoids | 4.20 | 609.18 | 40 |
| Cerrosillin | Flavonoids | 6.30 | 343.12 | 30 |
| Hesperetin-7-O-neohesperidoside | Flavonoids | 4.20 | 609.18 | -40 |
| Hesperetin-7-O-rutinoside | Flavonoids | 4.30 | 609.18 | -30 |
| L-Serine | Amino acids and derivatives | 0.70 | 106.05 | 20 |
| 6'-Hydroxy-3,4,2',3',4',5'-Hexamethoxychalcone | Flavonoids | 7.00 | 405.15 | 30 |
| Limonin | Terpenoids | 6.60 | 471.20 | 30 |
| Stachydrine | Alkaloids | 0.80 | 144.10 | 30 |
| 6'-Hydroxyjusticidin C | Lignans and Coumarins | 6.20 | 411.11 | 30 |
| 3',6'-Dihydroxy-3,4,2',4'-Tetramethoxychalcone | Flavonoids | 7.20 | 361.13 | 30 |
| Deacetylnomilin | Terpenoids | 6.40 | 473.22 | 30 |
| Gardenin C 5-[6''-(3-Hydroxy-3-Methylglutaryl)Glucoside]glucoside | Flavonoids | 4.60 | 873.27 | 30 |
| 5-Hydroxy-6,7,3',4'-tetramethoxyflavanone | Flavonoids | 7.20 | 361.13 | 30 |
| Ombuin 5-[6''-(3-Methylglutaconyl)Glucoside]Glucoside | Flavonoids | 4.10 | 781.22 | 30 |
| Quercetin 3,5,7,3'-tetramethyl ether | Flavonoids | 5.80 | 359.11 | 30 |
| 5,7-Dihydroxy-6,3',4',5'-tetramethoxyflavone | Flavonoids | 7.00 | 375.11 | 30 |
| 2,3,19-Trihydroxyolean-12-en-28-oic acid | Terpenoids | 7.70 | 489.36 | 30 |
| 7-Hydroxy-3,5,6,8-tetramethoxyflavone | Flavonoids | 5.50 | 359.11 | 30 |
| Obacunoic acid | Terpenoids | 6.50 | 473.22 | 30 |
| 4'-Hydroxy-5,6,7-trimethoxyflavone | Flavonoids | 5.90 | 329.10 | 30 |
| Trans-4-Hydroxycinnamic Acid Methyl Ester | Phenolic acids | 5.40 | 177.06 | -23 |
| 5,6,2',3',4',6'-Hexamethoxyflavone | Flavonoids | 6.40 | 403.14 | 30 |
| 5,7,8,3',4'-Pentamethoxyflavanone* | Flavonoids | 6.80 | 375.14 | 30 |
| 5'-Deoxy-5'-(methylthio)adenosine | Nucleotides and derivatives | 2.90 | 298.10 | 20 |
| 3',5-Dihydroxy-4',6,7-Trimethoxyflavanone | Flavonoids | 6.60 | 347.11 | 30 |
| 3,4-Methylenedioxy cinnamyl alcohol | Lignans and Coumarins | 5.40 | 177.06 | -30 |
| Nomilinic acid | Others | 6.70 | 531.23 | -30 |
| 3',4',5,6,7-Pentamethoxyflavanone* | Flavonoids | 6.70 | 375.14 | 30 |
| Synephrine | Alkaloids | 0.80 | 168.10 | 30 |
| Homoeriodictyol | Flavonoids | 5.90 | 303.09 | 40 |
| Apigenin-7-O-rutinoside (Isorhoifolin) | Flavonoids | 4.00 | 579.17 | 30 |
| Desmethylsinensetin 5-[6''-(3-Hydroxy-3-Methylglutaryl)Glucoside] | Flavonoids | 3.90 | 665.21 | 30 |
| Quercetin-3-O-(2''-O-Rhamnosyl)rutinoside | Flavonoids | 3.40 | 757.22 | 30 |
| Jasminoside A | Others | 3.30 | 331.18 | 30 |
| Picrocrocin | Others | 3.20 | 331.18 | 30 |
